# Longitudinal analysis of symptoms and healthcare utilization among daily cannabis-using persons living with HIV: impact of co-occurring cocaine use

**DOI:** 10.1186/s42238-026-00443-7

**Published:** 2026-05-01

**Authors:** Valden A. Denha, Leslie H. Lundahl, Jonathan A. Cohn, Mark K. Greenwald, Eric A. Woodcock

**Affiliations:** 1 Department of Population and Health Sciences, School of Public Health, University of Michigan, Ann Arbor, MI USA; 2 Irvin D. Reid Honors College, College of Liberal Arts and Sciences, Wayne State University, Detroit, MI USA; 3 Department of Psychiatry and Behavioral Neurosciences, Wayne State University School of Medicine, Detroit, MI 48201 USA; 4 Department of Internal Medicine, Wayne State University School of Medicine, Detroit, MI USA; 5 Department of Pharmacy Practice, Eugene Applebaum College of Pharmacy and Health Sciences, Wayne State University, Detroit, MI USA; 6 Department of Pharmacology, Wayne State University School of Medicine, Detroit, MI USA

**Keywords:** HIV, Cannabis, Cocaine, Polysubstance Use, Healthcare Utilization

## Abstract

**Background:**

Cannabis use is common among persons living with human immunodeficiency virus (HIV, PLWH) and has been associated with disrupted sleep, psychiatric symptoms, and increased healthcare utilization. However, the influence of concurrent cocaine and cannabis use on symptom trajectories and healthcare engagement remains unclear.

**Methods:**

One hundred nineteen PLWH who reported daily cannabis use were assessed longitudinally: baseline, 3-month, and 6-month follow-up. Participants were stratified into concurrent cocaine users vs. non-users based on urine drug screen results (Coc + *n* = 34 vs. Coc- *n* = 85). Assessments included substance-use characteristics, viral load, healthcare utilization (HIV-specific vs. non-specific medical visits), HIV-related symptoms (poor sleep, fatigue, pain, poor appetite), and psychiatric symptoms (depression, anxiety).

**Results:**

From baseline to 6-month follow-up, HIV clinic visit frequency declined 46.7% (*p* = 0.002), which was predicted by less-frequent baseline cannabis use (*p* = 0.03). Poor sleep, fatigue, and poor appetite reliably correlated with psychiatric symptom severity (*r*s = 0.23–0.57), whereas pain severity was most closely linked to non-HIV-specific healthcare utilization (*r*s = 0.23–0.36). Cocaine co-use was associated with greater non-HIV-specific healthcare utilization, worse sleep, and less-frequent cannabis use (*p*s < 0.05).

**Conclusions:**

Among daily cannabis-using PLWH, less-frequent cannabis use at study baseline predicted a 47% decline in HIV clinic visits suggesting that frequent cannabis use (or associated factors) may perpetuate continued HIV healthcare utilization. Cocaine co-use was associated with greater healthcare utilization, worse sleep quality, and less-frequent cannabis use, but did modulate self-reported depression or anxiety symptoms. In sum, our findings show a complex interplay between longitudinal patterns of cannabis use, somatic and psychiatric symptom severity, and healthcare utilization among PLWH.

**Trial registration:**

This study was registered at ClinicalTrials.gov (NCT01536899).

**Supplementary Information:**

The online version contains supplementary material available at 10.1186/s42238-026-00443-7.

## Introduction

Persons living with human immunodeficiency virus (HIV; PLWH) comprise ~ 1.2 million individuals in the United States (US) (HIV.gov n.d.). PLWH often experience significant challenges managing somatic and psychiatric symptoms while maintaining antiretroviral treatment (ART) adherence (Kipp et al. 2017). Managing HIV symptoms imposes considerable expenses on the healthcare system, with lifetime medical costs estimated at $420,285 per PLWH in 2019 US dollars (estimated range: $326,411–$490,045) (Chesson et al. 2021). Further, as ARTs have become increasingly effective, PLWH are living longer (Trickey et al. 2023) which underscores the importance of effective symptom management to minimize healthcare and economic burdens.

Cannabis use is prevalent among PLWH, with self-reported past-month rates as high as 25%, often driven by the perception that cannabis can alleviate HIV symptoms such as pain, fatigue, poor sleep, and appetite loss (Andreae et al. 2015, Greenwald et al. 2023, Wrona et al. 2025). Interviews with PLWH have shown that many describe cannabis as helpful for managing pain, appetite, anxiety, and sleep difficulties, though some also express uncertainty about its effects (Costiniuk et al. 2019). Although patients commonly report using cannabis to manage chronic pain, its efficacy is questionable (Greenwald 2025), and chronic pain is often associated with concurrent use of prescription opioids and other substances (Perron et al. 2015). Although some individuals report symptom relief, particularly for appetite stimulation and weight maintenance, cannabis use has also been associated with higher rates of psychiatric symptoms including depression and anxiety (Wrona et al. 2025, Montgomery et al. 2019). These psychiatric symptoms not only co-occur with substance use but also serve as risk factors for its initiation and progression. Recent network analysis has further shown that sadness, pessimism, and worthlessness are core symptoms in PLWH, underscoring the importance of addressing affective distress in this population (Han et al. 2023). For instance, epidemiological research has shown that adolescents with mood, anxiety, and behavioral disorders are significantly more likely to begin using substances and develop substance use disorders, including cannabis use disorder (Swendsen et al. 2010). Additionally, nearly half of individuals with cannabis use disorder have co-occurring psychiatric diagnoses (Gorelick 2023). Somatic symptoms, including pain and fatigue, are also closely linked to psychiatric distress in PLWH, including depression symptoms (Ferrando et al. 1998, Scott et al. 2018).

The confluence of these factors can reduce treatment adherence and increase healthcare utilization. Cannabis-related emergency department visits have increased significantly in recent years, with rates rising by double digits through 2018, consistent with an upward trend continuing into the 2020 s (Roehler et al. 2022). Approximately one-quarter to one-third of these visits involve acute psychiatric symptoms such as anxiety, psychosis, and suicidal ideation (Shelton et al. 2019, Crocker et al. 2021). Conversely, another study found no relationship between cannabis-use frequency and healthcare utilization or adverse health outcomes in a primary care sample (Fuster et al. 2014). Among PLWH, daily cannabis use has been associated with missed HIV care appointments (Kipp et al. 2017, Wagner et al. 2011), suggesting lower treatment adherence. Taken together, relationships between cannabis use, symptom severity, and healthcare engagement are complex and not well-understood, especially among PLWH.

Among PLWH, psychostimulant use, particularly cocaine, is common and may compound health risks. Psychostimulant (cocaine and/or methamphetamine) use has been associated with worse ART adherence, increased symptom burden and healthcare utilization, and faster HIV disease progression and mortality (Crocker et al. 2021, Carrico et al. 2014, Lee et al. 2023). In non-HIV populations, cocaine use has been shown to impact sleep, appetite, and pain perception (Angarita et al. 2016, Compton 1994), and cocaine + cannabis polysubstance use has been linked to increased psychiatric symptom severity, including higher rates of psychosis and suicidality (Daldegan-Bueno et al. 2021, Gonzalez-Pinto et al. 2009). However, among PLWH specifically, the impact of polysubstance use on psychiatric symptoms and healthcare utilization remains poorly understood and understudied.

This prospective observational study aimed to address these knowledge gaps by examining longitudinal patterns of cannabis-use frequency, HIV-related symptom severity, psychiatric symptom severity, and their associations with healthcare utilization in PLWH who use cannabis daily. Further, we aimed to investigate the specific impact of co-occurring use of psychostimulants (cocaine in this sample) on these relationships. We hypothesized that cannabis-use frequency would positively correlate with HIV-related symptom severity which, in turn, would positively correlate with psychiatric symptom severity and healthcare utilization. Separately, we hypothesized that co-occurring cocaine and cannabis use would predict greater HIV-related somatic and psychiatric symptom burden and healthcare utilization, relative to those who did not use cocaine. These hypotheses reflect the tight coupling of somatic and psychiatric symptoms in PLWH, and the potential exacerbating influence of polysubstance use on symptom burden and care engagement (Kipp et al. 2017, Crocker et al. 2021, Lee et al. 2021).

## Methods

### Participants

Study procedures were approved by the Wayne State University Institutional Review Board (ClinicalTrials.gov: NCT01536899). Between May 2012 and April 2015, adults living with HIV, aged 18 to 70 years, were recruited for this study via the university’s affiliated Infectious Disease Clinic, with no restrictions based on race, ethnicity, or biological sex. The clinic maintains a caseload of ~ 2,000 patients living with HIV; of whom, most are male (~ 73%) and Black/African-American (~ 87%), and most self-report substance use (tobacco use being most common). Three methods of participant recruitment were used. First, IRB-approved flyers and brochures posted in the clinic (waiting room, procedure rooms) provided basic study information and encouraged interested individuals to contact research staff. Second, based on review of medical records and encounters with patients, clinic staff cooperated by identifying potentially interested candidates, providing them with a flyer or brochure, and referring them to research staff. Third, we encouraged word-of-mouth referral from participants to other potential candidates in the clinic (there was no financial incentive to refer).

Prospective subjects were invited to participate if they self-reported an HIV diagnosis and daily cannabis use. After verification of no recent alcohol consumption (breath alcohol ≤ 0.02%), volunteers provided written informed consent. Candidates were included in this study if they self-reported daily cannabis use over the past 90 days (or longer), screened urine-positive for Δ−9-tetrahydrocannabinol (THC ≥ 50ng/mL), exhibited normative verbal intelligence (Shipley Institute of Living Score ≥ 80) (Zachary 1991), and reported no history of neurological disease or injury. Other substance use, including cocaine, was allowed, whereas pregnant females (urine human chorionic gonadotropin test) were excluded.

Each study visit lasted about 3–4 h, during which participants completed a battery of subjective and behavioral assessments, as well as provided a urine sample tested for THC (≥ 50ng/mL), morphine (≥ 300ng/ml), benzoylecgonine (≥ 150ng/ml), meth/amphetamine (≥ 1000ng/ml), barbiturates (≥ 300ng/ml), and benzodiazepines (≥ 300ng/ml). Participants completed assessments every three months for one year, i.e., five assessments; however, analyses herein were restricted to the first three study visits, i.e., ‘baseline’, 3-months, and 6-months post-baseline, to investigate near-term relationships. Participants were compensated $100 for completing the baseline visit and $40 for each follow-up visit via prepaid debit card. Throughout this study, cannabis use for medical (but not yet recreational) purposes was legal in the State of Michigan under the Michigan Medical Marihuana Act (Michigan Legislature 2008).

### Baseline assessments

The medical and substance use history questionnaires described below were administered at the ‘baseline’ visit only.

#### Medical history

Participants self-reported the year of HIV diagnosis, from which years living with HIV was calculated by subtracting the diagnosis year from study intake year. Pre-study viral load (copies/ml) and CD4 cell count (cells/mm^3^) were extracted from the patient’s electronic medical record. Other medical conditions and diagnoses were assessed, but analyses focused on HIV chronicity.

#### Substance use history

Substance use history was assessed with a self-report questionnaire tailored from the Drug History and Use Questionnaire (Moses and Greenwald 2019). Participants provided detailed information about their use of tobacco, alcohol, cannabis, cocaine, meth/amphetamine (prescribed and nonmedical), sedatives (prescribed and nonmedical), and opioids (prescribed and nonmedical). For each substance, participants reported the age at first use, the age at first regular use (at least once per week), and the age at first daily use. For cannabis, duration of use was calculated by subtracting the age at first use from the participant’s current age. A single item asked subjects to estimate the percentage of time using cannabis for symptom relief, i.e., medicinal use (0–100%).

### Longitudinal assessments

The measures below were administered at each study visit to assess longitudinal changes.

#### Cannabis-use frequency

Past 90-day cannabis-use frequency was calculated by multiplying the mean number of cannabis uses per day over the past week by number of days using over the past 30 days and scaled by 3 (months).

#### HIV-related somatic symptoms

Participants self-reported the severity of several common HIV-related somatic symptoms, including ‘poor appetite’, ‘fatigue or low energy’, ‘pain’, and ‘poor sleep’. Participants rated the severity of each symptom over the past 90 days on a Likert scale from 0 to 3 (0 = not at all, 1 = slightly, 2 = somewhat, 3 = severe).

#### Psychiatric symptoms

Recent depression and anxiety symptoms were assessed using the Beck Depression Inventory-II (BDI-II) (Beck et al. 1996) and State-Trait Anxiety Inventory (STAI) (Spielberger 1989), The BDI-II assesses past two-week severity of neurovegetative symptoms of depression across 21 items using a Likert scale (0=’symptom absent’, 3=’symptom is severe’) for the past two-weeks (total: 0–63; higher scores indicate more severe depression symptoms). A score of 14 is considered the threshold for ‘mild’ depressive symptom severity. Here we report data from the 20-item state-version of the STAI, which reflects how anxious someone feels in the moment, rated on a scale from 1 to 4, where 1 reflects “not at all” and 4 reflects “very much so.” Scores for each subscale range from 20 to 80, with higher scores indicating greater anxiety. Here, analyses focused on the ‘state’ anxiety subscale to capture fluctuations in time.

#### Healthcare utilization

Participants responded to five questions regarding their healthcare utilization over the previous 3 months. Four questions were summed to create a composite variable which reflects non-HIV-specific care, ‘Healthcare Clinic Visits’: “How many times have you been admitted to the hospital in the past 3 months?”; “How many times have you visited the ER in the past 3 months?”; “How many times have you visited urgent care in the past 3 months?”; and “How many times have you seen a general healthcare provider in the past 3 months?”. The final question, “How many times have you been admitted to an HIV clinic in the past 3 months?”, was analyzed in isolation (‘HIV Clinic Visits’).

### Participant groups

PLWH were sub-categorized by co-occurring cocaine use. Participants whose urine samples screened positive for cocaine metabolites at baseline, 3-, or 6-month follow-up (urine benzoylecgonine ≥ 150 ng/ml) were categorized as ‘cocaine-positive’ (‘Coc+’; *n* = 34), whereas participants whose urine samples tested negative for cocaine metabolites at all timepoints were deemed ‘cocaine-negative’ (‘Coc-’; *n* = 85). Of note, participants were urine screened for meth/amphetamine use at each timepoint, but no samples tested positive (≤ 1000ng/ml) which is consistent with substance use epidemiological monitoring data for Detroit at the time this study was conducted (Arfken 2018). Cocaine use, in contrast, was detected and used as a grouping variable in analyses, and urinalysis results aligned well with self-reported cocaine use.

### Statistical analyses

Statistical analyses were conducted using SPSS software v29 (IBM; Armonk, NY). Data were first examined for missing values and normality using skewness and kurtosis statistics and the Shapiro-Wilk test. Non-normal distributions were normalized using statistical transformations. Group effects for nominal/categorical variables were evaluated using chi-square tests of independence and one-way Analyses of Variance or Covariance (ANOVA or ANCOVA) for continuous/ordinal variables, controlling for age (which differed between cocaine cohorts). Paired t-tests were used to evaluate within-subject changes over time. This piecemeal analytic approach was employed to minimize the impact of missing data at the 3-month timepoint while facilitating evaluation of subtle longitudinal changes preserving the 90-day temporal resolution. For Group and Time effects, the threshold for statistical significance was *p* ≤ 0.05 after Benjamini-Hochberg False Discovery Rate (FDR) correction for multiple comparisons (i.e., *p*_FDR_) (Benjamini and Hochberg 1995) within each symptom category and timepoint. Bivariate Pearson correlations evaluated linear relationships among variables within each cocaine cohort (and for completeness, the overall sample; see Supplemental Material). Raw Pearson *r* values are depicted for statistically significant bivariate relationships after Benjamini-Hochberg FDR correction, i.e., *p*_FDR_≤0.05 (Benjamini and Hochberg 1995). Finally, stepwise linear regression analyses investigated predictors of the change in healthcare utilization from baseline to 6-month follow-up, including substance use characteristic, demographic variables, pre-study viral load and CD4 cell count, and baseline and 3-month follow-up measures of cannabis-use frequency, somatic symptoms, and psychiatric symptoms. For descriptive purposes, means (M) plus/minus one standard deviation (SD) or percentage endorsed (%) are presented. Data completeness at each timepoint is noted in the Tables.

## Results

### Participant characteristics

The sample consisted of 119 PLWH who reported daily cannabis use during the 90 days prior to the baseline visit. Most participants were male (63.0%) and African American (89.1%) with a mean age of 45.2 (9.7) years (Table 1). UDS testing revealed modest recent drug use: 1.3% UDS + for barbiturates, 1.0% UDS + for methadone, 13.3% UDS + for morphine, and 13.6% UDS + for benzodiazepines (UDS+ prevalence did not differ between groups: *p*s > 0.05). On average, participants initiated regular cannabis use around age 20 and transitioned to daily cannabis use around age 22. Participants reported living with an HIV diagnosis for 11.3 (7.4) years.

**Table 1:** Sample characteristics

	Overall (*N* = 119)	Coc+ (*n* = 34)	Coc- (*n* = 85)	*p*
Age (yrs)	44.8 (9.7)	**49.4 (5.3)**	**43.0 (10.5)**	**0.01**
Sex (% Male)	63.6%	61.8%	64.3%	0.80
Race (% Black/African American)	90.6%	97.1%	88.0%	0.13
Age at First Cocaine Use (yrs)	24.6 (6.5)	24.3 (7.3)	24.8 (6.0)	0.75
Age at Regular MJ Use (yrs; 3x/wk)	20.0 (7.2)	18.3 (6.1)	20.7 (7.5)	0.11
Age at Daily MJ Use (yrs)	22.0 (9.3)	23.3 (10.1)	22.7 (9.1)	0.77
Cannabis Use for Symptom Relief (% Time)	59.5%	58.5%	59.9%	0.85
Viral Load (Log_10_(copies/ml))	2.4 (1.4)	2.4 (1.3)	2.4 (1.4)	0.89
VL Suppressed (% <200 copies/ml)	74.1%	70.0%	75.9%	0.81
CD4 Count (cells/mm^3^)	539.2 (309.3)	500.2 (315.9)	551.0 (309.2)	0.56
Years Since HIV + Dx (yrs)	11.3 (7.4)	12.8 (8.6)	10.7 (6.7)	0.18

Mean (± 1 Standard Deviation) or % endorsed are depicted. Significant group differences noted in bold. *VL* Viral Load. VL/CD4 assayed at, or prior to, baseline study visit (data available in subset of sample; *n* = 81)

The Coc + and Coc- groups did not differ (*p*s > 0.10) for pre-study viral load, CD4 cell count, years living with an HIV diagnosis, cannabis-use frequency, and demographic characteristics, except for age. The Coc+ group was significantly older than the Coc- group; thus, between-group analyses included age as a covariate.

The Coc+ group reported an average age at first cocaine use of 24.3 (7.3) years old. On average, the Coc+ group reported using cocaine approximately weekly (1–4 times per month) which remained stable throughout the study (*p*s > 0.40). None of the Coc+ group reported meth/amphetamine use and no subject in either group exhibited a UDS + for meth/amphetamine at any timepoint. As stated in the Methods, each subject in the Coc- group exhibited UDS- for cocaine and meth/amphetamine at every timepoint.

### FDR-corrected group comparisons

#### Baseline assessment

At baseline, the Coc+ group reported worse sleep quality than the Coc- group with mean severity scores of 1.9 (1.1) vs. 1.3 (1.2), *F*(1) = 6.93, *p*_FDR_=0.04, controlling for age (Table 2). Relative to the Coc- group, the Coc+ group also reported 2-fold more non-HIV-specific healthcare visits (‘Healthcare Clinic Visits’), *F*(1,117) = 7.46, *p*_FDR_=0.02, and 40% more HIV clinic visits, *F*(1,117) = 4.85, *p*_FDR_*=*0.02.

**Table 2:** Cross sectional analyses

Baseline Assessment				
	Overall (*N* = 119)	Coc+ (*n* = 34)	Coc- (*n* = 85)	*p*_FDR_
Past 90-day Cannabis Use Freq (#)	250.7 (208.1)	193.2 (150.3)	274.0 (224.0)	-
* Past 90-day Healthcare Utilization*				
Healthcare Clinic Visits (#)	1.5 (2.2)	**2.5 (3.4)**	**1.2 (1.8)**	**0.02**
HIV Clinic Visits	1.5 (1.5)	**2.0 (2.0)**	**1.4 (1.2)**	**0.02**
* Past 90-day Symptom Severity (0–3)*				
Poor Appetite	1.2 (1.1)	1.0 (1.1)	1.3 (1.1)	-
Fatigue/Low Energy	1.3 (1.1)	1.2 (1.1)	1.4 (1.2)	-
Pain	1.8 (1.1)	1.9 (1.1)	1.8 (1.1)	-
Poor Sleep	1.5 (1.2)	**1.9 (1.1)**	**1.3 (1.2)**	**0.04**
* Psychiatric Symptom Severity*				
Depression (BDI-II)	15.0 (10.1)	16.9 (8.9)	14.3 (10.6)	-
Anxiety (STAI; *state*)	41.7 (11.6)	43.4 (10.3)	41.1 (12.1)	-

3-Month Assessment				
	Overall (*N* = 97)	Coc+ (*n* = 25)	Coc- (*n* = 72)	*p*_FDR_
Past 90-day Cannabis Use Freq (#)	207.5 (166.6)	158.5 (153.9)	224.5 (168.4)	-
* Past 90-day Healthcare Utilization*				
Healthcare Clinic Visits (#)	1.0 (1.7)	1.3 (1.7)	0.9 (1.7)	-
HIV Clinic Visits	1.3 (1.8)	1.3 (1.3)	1.2 (1.9)	-
* Past 90-day Symptom Severity (0–3)*				
Poor Appetite	1.0 (1.0)	1.1 (1.1)	1.0 (1.0)	-
Fatigue/Low Energy	1.1 (1.1)	1.2 (1.1)	1.1 (1.2)	-
Pain	1.7 (1.2)	1.9 (1.2)	1.7 (1.3)	-
Poor Sleep	1.3 (1.1)	1.4 (1.1)	1.3 (1.2)	-
* Psychiatric Symptom Severity*				
Depression (BDI-II)	14.4 (10.7)	14.4 (10.6)	14.4 (10.8)	-
Anxiety (STAI; *state*)	41.4 (12.0)	41.2 (11.2)	41.5 (12.4)	-

6-Month Assessment				
	Overall (*N* = 119)	Coc+ (*n* = 28)	Coc- (*n* = 69)	*p*_FDR_
Past 90-day Cannabis Use Freq (#)	221.7 (184.1)	**150.0 (144.4)**	**250.7 (191.4)**	**0.04**
* Past 90-day Healthcare Utilization*				
Healthcare Clinic Visits (#)	1.5 (2.3)	**2.6 (3.3)**	**1.0 (1.5)**	**0.02**
HIV Clinic Visits	0.8 (0.7)	0.8 (0.7)	0.8 (0.7)	-
* Past 90-day Symptom Severity (0–3)*				
Poor Appetite	0.9 (1.0)	1.0 (1.2)	0.9 (1.0)	-
Fatigue/Low Energy	1.2 (1.1)	1.3 (1.1)	1.1 (1.1)	-
Pain	1.6 (1.3)	1.5 (1.4)	1.7 (1.2)	-
Poor Sleep	1.3 (1.2)	1.5 (1.2)	1.2 (1.2)	-
* Psychiatric Symptom Severity*				
Depression (BDI-II)	14.3 (11.0)	16.7 (11.2)	13.3 (10.8)	-
Anxiety (STAI; *state*)	41.4 (11.9)	41.8 (10.7)	41.3 (12.5)	-

Mean (± 1 SD) depicted. False Discovery Rate (FDR; Benjamini-Hochberg) correction applied

#### 3-month assessment

At the 3-month follow-up, no statistically significant group differences emerged (*p*_FDR_>0.05).

#### 6-month assessment

At the 6-month follow-up, past 90-day cannabis-use frequency was 40% lower in the Coc+ group compared to the Coc- group, controlling for age, *F*(1,93) = 4.60, *p*_FDR_=0.04, by ~ 100 uses or ~ 1 use per day [150.0 (144.4) vs. 250.7 (191.4), respectively]. Conversely, the Coc+ group reported 1.6-fold more non-HIV-specific healthcare visits compared to the Coc- group, controlling for age, [2.6 (3.3) vs. 1.0 (1.5), respectively], *F*(1,94) = 9.56, *p*_FDR_=0.02.

### FDR-corrected Pearson correlations

In the Coc- cohort (Table 3), non-HIV-specific healthcare utilization significantly correlated with fatigue and depression symptom severity at the 3-month follow-up (*r*s = 0.32–0.36), and pain at the 6-month follow-up (*r* = 0.36). At the 3-month follow-up, HIV healthcare visits correlated with depression symptom severity (*r* = 0.36). Across time points, somatic symptoms were inter-related (as expected), and tended to correlate with depression and anxiety severity, with the notable exception of pain, which exhibited a significant relationship with depression (and not anxiety) severity at baseline only.

**Table 3:** FDR-corrected Pearson correlations among non-cocaine-using individuals

Coc- (*n*=85): Baseline Assessment									
		2	3	4	5	6	7	8	9
1	Past 90-day Cannabis Use Frequency (#)	-	-	-	-	-	-	-	-
	*Past 90-day Healthcare Utilization*								
2	Healthcare Clinic Visits (#)	-	-	-	-	-	-	-	-
3	HIV Clinic Visits	-	-	-	-	-	-	-	-
	*Past 90-day Symptom Severity (0–3)*								
4	Poor Appetite	-	-	-	0.35*	0.40*	-	0.44*	0.43*
5	Fatigue/Low Energy	-	-	-	-	0.44*	0.28*	0.57*	0.45*
6	Pain	-	-	-	-	-	-	0.29*	-
7	Poor Sleep	-	-	-	-	-	-	0.37*	-
	*Psychiatric Symptom Severity*								
8	Depression (BDI-II)	-	-	-	-	-	-	-	0.80*
9	Anxiety (STAI;*state*)	-	-	-	-	-	-	-	-

Coc- (*n*=72): 3-Month Assessment									
		2	3	4	5	6	7	8	9
1	Past 90-day Cannabis Use Frequency (#)	-	-	-	-	-	-	-	-
	*Past 90-day Healthcare Utilization*								
2	Healthcare Clinic Visits (#)	-	-	-	0.36*	-	-	0.32*	-
3	HIV Clinic Visits	-	-	-	-	-	-	0.36*	-
	*Past 90-day Symptom Severity (0–3)*								
4	Poor Appetite	-	-	-	0.35*	-	-	0.32*	0.40*
5	Fatigue/Low Energy	-	-	-	-	0.43*	0.54*	0.57*	0.50*
6	Pain	-	-	-	-	-	-	-	-
7	Poor Sleep	-	-	-	-	-	-	0.40*	0.34*
	*Psychiatric Symptom Severity*								
8	Depression (BDI-II)	-	-	-	-	-	-	-	0.81*
9	Anxiety (STAI;*state*)	-	-	-	-	-	-	-	-

Coc- (*n*=69): 6-Month Assessment									
		2	3	4	5	6	7	8	9
1	Past 90-day Cannabis Use Frequency (#)	-	-	-	-	-	-	-	-
	*Past 90-day Healthcare Utilization*								
2	Healthcare Clinic Visits (#)	-	-	-	-	0.36*	-	-	-
3	HIV Clinic Visits	-	-	-	-	-	-	-	-
	*Past 90-day Symptom Severity (0–3)*								
4	Poor Appetite	-	-	-	0.39*	-	-	-	-
5	Fatigue/Low Energy	-	-	-	-	-	0.43*	0.61*	0.58*
6	Pain	-	-	-	-	-	-	-	-
7	Poor Sleep	-	-	-	-	-	-	0.49*	0.40*
	*Psychiatric Symptom Severity*								
8	Depression (BDI-II)	-	-	-	-	-	-	-	0.79*
9	Anxiety (STAI; *state*)	-	-	-	-	-	-	-	-

Raw Pearson *r* values are shown whereas statistical significance evaluated after False Discovery Rate (FDR; Benjamini-Hochberg) correction: *p<0.05, **p<0.01, ***p<0.001

In the Coc+ cohort (Table 4), correlations followed a similar pattern, but significant relationships were more sparse, likely due to limited statistical power (*n*s = 25–34). Non-HIV-specific healthcare utilization correlated with fatigue severity at 3-month follow-up (*r* = 0.57) and pain severity at 6-month follow-up (*r* = 0.52). HIV clinic utilization correlated with poor appetite at baseline only (*r* = 0.56). Depression symptom severity correlated with poor sleep at baseline and 6-month follow-up (*r*s = 0.44–0.47), and anxiety severity across time points (*r*s = 0.49–0.61). Somatic symptoms were inter-correlated at 6-month follow-up (*r*s = 0.45–0.67) but not at baseline or 3-month follow-up.

**Table 4:** FDR-corrected Pearson correlations among cocaine-using individuals

Coc+ (*n* = 34): Baseline Assessment									
		2	3	4	5	6	7	8	9
1	Past 90-day Cannabis Use Frequency (#)	-	-	-	-	-	-	-	-
	*Past 90-day Healthcare Utilization*								
2	Healthcare Clinic Visits (#)	-	-	-	-	-	-	-	-
3	HIV Clinic Visits	-	-	0.56*	-	-	-	-	-
	*Past 90-day Symptom Severity (0–3)*								
4	Poor Appetite	-	-	-	-	-	-	-	-
5	Fatigue/Low Energy	-	-	-	-	-	-	-	-
6	Pain	-	-	-	-	-	-	-	-
7	Poor Sleep	-	-	-	-	-	-	0.47*	-
	*Psychiatric Symptom Severity*								
8	Depression (BDI-II)	-	-	-	-	-	-	-	0.61*
9	Anxiety (STAI; *state*)	-	-	-	-	-	-	-	-

Coc+ (*n* = 25): 3-Month Assessment									
		2	3	4	5	6	7	8	9
1	Past 90-day Cannabis Use Frequency (#)	-	-	-	-	-	-	-	-
	*Past 90-day Healthcare Utilization*								
2	Healthcare Clinic Visits (#)	-	-	-	0.57*	-	-	-	-
3	HIV Clinic Visits	-	-	-	-	-	-	-	-
	*Past 90-day Symptom Severity (0–3)*								
4	Poor Appetite	-	-	-	-	-	-	-	-
5	Fatigue/Low Energy	-	-	-	-	-	-	-	-
6	Pain	-	-	-	-	-	-	-	-
7	Poor Sleep	-	-	-	-	-	-	-	-
	*Psychiatric Symptom Severity*								
8	Depression (BDI-II)	-	-	-	-	-	-	-	0.60*
9	Anxiety (STAI; *state*)	-	-	-	-	-	-	-	-

Coc+ (*n*=28): 6-Month Assessment									
		2	3	4	5	6	7	8	9
1	Past 90-day Cannabis Use Frequency (#)	-	-	-	-	-	-	-	-
	*Past 90-day Healthcare Utilization*								
2	Healthcare Clinic Visits (#)	-	-	-	-	0.52*	-	-	-
3	HIV Clinic Visits	-	-	-	-	-	-	-	-
	*Past 90-day Symptom Severity (0–3)*								
4	Poor Appetite	-	-	-	0.65*	-	-	-	-
5	Fatigue/Low Energy	-	-	-	-	-	0.67*	-	-
6	Pain	-	-	-	-	-	0.58*	-	-
7	Poor Sleep	-	-	-	-	-	-	0.44*	-
	*Psychiatric Symptom Severity*								
8	Depression (BDI-II)	-	-	-	-	-	-	-	0.49*
9	Anxiety (STAI; *state*)	-	-	-	-	-	-	-	-

Raw Pearson *r* values are shown whereas statistical significance evaluated after False Discovery Rate (FDR; Benjamini-Hochberg) correction: *p < 0.05, **p < 0.01, ***p < 0.001

For completeness, full sample bivariate correlations are shown in Supplemental Table 1.

### FDR-corrected longitudinal analyses

The Coc- and Coc+ groups did not differ in percentage rates of 3-month and 6-month follow-up, with ~ 20% attrition relative to baseline. Overall, past 90-day cannabis-use frequency declined significantly from baseline to 3-month follow-up, *t*(96) = 1.98, *p* = 0.04, i.e., Time effect, and there were no significant group differences (Supplemental Table 2). Across groups, non-HIV-specific healthcare visits remained stable across time (*p*_FDR_≥0.10), whereas HIV clinic visits nominally decreased across all time frames (*p*_FDR_<0.10) and significantly decreased from baseline to 6-month follow-up by 46.7% (*p*_FDR_=0.002). Psychiatric symptom severity and somatic symptoms were unchanged across all time points (*p*_FDR_≥0.05).

### Stepwise linear regression analyses

Stepwise linear regression analyses investigated predictors of the significant reduction in HIV clinic visits of 46.7% from baseline to the 6-month follow-up (*p*_FDR_=0.002) and revealed one significant predictor: baseline cannabis-use frequency [*F*(1,84) = 4.80, *p* = 0.03; *β* = 0.99, *R*^*2*^ = 0.05]. The decline in HIV clinic visits was associated with less-frequent cannabis use at baseline, suggesting that more-frequent cannabis use may perpetuate sustained HIV-related healthcare engagement among PLWH.

## Discussion

This prospective observational study examined relationships between self-reported cannabis-use frequency, HIV-related somatic symptoms, psychiatric symptoms, and healthcare utilization among daily cannabis-using PLWH. Cross-sectional analyses indicated that co-occurring cocaine use (broadly defined as any UDS-verified cocaine use from baseline through 6-month follow-up) was associated with greater healthcare utilization, especially non-HIV-specific clinic visits, worse sleep quality at baseline, and less-frequent cannabis use at 6-month follow-up, compared to non-cocaine users. Pain severity and fatigue were most closely associated with non-HIV-specific healthcare utilization, whereas HIV clinic visits were associated with depression severity and poor appetite. Poor sleep, fatigue, and poor appetite (each inter-related) were most closely associated with depression and anxiety symptom severity, particularly among the Coc- cohort, whereas pain less so. Longitudinal analyses across the 3- and 6-month follow-up assessments indicated that cannabis-use frequency significantly declined over time, whereas somatic and psychiatric symptoms were generally stable and of ‘mild’-to-‘moderate’ severity. Finally, non-HIV-specific healthcare visits remained stable over time, whereas HIV clinic visits significantly declined by 46.7% from baseline to 6-month follow-up. Stepwise linear regression analyses indicated less-frequent cannabis use at baseline predicted a greater decline in HIV clinic visits from baseline to 6-month follow-up, suggesting that more-frequent cannabis use may perpetuate continued HIV-related healthcare utilization (Becker et al. 2022).

In contrast with prior research (Kipp et al. 2017), our findings indicate less-frequent baseline cannabis use predicted a nearly 50% decline in HIV-related healthcare utilization by the 6-month follow-up, which has economic and healthcare system implications. Interestingly, the decline in HIV clinic visits was not predicted by baseline viral load or CD4 cell counts and was not paralleled by changes in self-reported somatic or psychiatric symptom severity, which remained stable across time and were ‘mild’-to-‘moderate’ in severity. The lack of coupling between symptom severity and HIV clinic visits is noteworthy and underscores the complexity of these relationships. Whereas other studies have shown acute symptom relief immediately following cannabis use (Stith et al. 2018), our longitudinal data generally do not show symptom relief on a longer time horizon (e.g., months) (Becker et al. 2022, Haller 2024). Further, the converse was also not supported by our data. Cannabis-use frequency significantly declined from baseline to 3-month follow-up. Yet, there was no evidence of a subsequent increase or ‘unmasking’ of somatic or psychiatric symptoms, nor an increase in healthcare utilization, during that period (nor from 3-to-6-month follow-up period). Finally, cross-sectional analyses did not identify significant associations between cannabis-use frequency and somatic or psychiatric symptom severity at any timepoint. From a harm reduction and patient perspective, our data suggest that PLWH may be able to reduce their cannabis-use frequency without compensatory increases in symptom severity or healthcare utilization. Future research is needed to determine whether factors such as cannabis-use patterns, THC: cannabidiol ratios, or individual differences in endocannabinoid system signaling moderate the therapeutic potential of cannabis (Lee et al. 2021, Cservenka et al. 2018). THC potency and route of cannabis administration were not assayed in this study, which may have influenced symptom expression and limited our ability to fully characterize cannabis effects. Finally, qualitative or mixed-methods research may be particularly useful for understanding patient lived experiences, attitudes toward, and perceived effectiveness of, cannabis as a therapeutic (Greenwald et al. 2023), and other important factors not captured by the surveys administered.

Our findings show that pain severity and fatigue were most consistently associated with non-HIV-specific healthcare utilization (though significant correlations were sparse). Poor sleep, fatigue, and poor appetite were closely linked to self-reported depression and anxiety symptom severity whereas pain was generally not correlated with depression or anxiety symptom severity. Our observations are consistent with evidence that affective symptoms organize broader patterns of distress in PLWH (Han et al. 2023). This dissociation may help physicians to understand factors that drive healthcare utilization vs. vulnerability factors related to psychiatric distress among PLWH. From the patient’s perspective, effective management of these symptoms may substantially improve quality of life. Prior studies have shown that pain and poor appetite are common and burdensome in PLWH and may contribute to increased healthcare utilization (Ferrando et al. 1998, Barroso et al. 2010), whereas fatigue has been linked to functional impairment and psychological distress (Barroso et al. 2010, Irwin et al. 2018). Prior research has shown that more than half of PLWH report significant fatigue, often related to anemia, depression, and physical inactivity (Gebreyesus et al. 2020). Further, poor diet and nutrient deficiencies among individuals who use drugs, including cannabis and psychostimulants, may contribute to symptoms such as poor appetite and fatigue in this population (Mahboub et al. 2020). Future studies are needed to evaluate interventions to alleviate somatic symptoms as well as psychiatric symptoms, healthcare visits, and improve quality of life in this population (Ferrando et al. 1998, Barroso et al. 2010). Finally, it is unclear how structural barriers to care, stigma, or other socioeconomic factors modulated these relationships in this sample.

Co-occurring cocaine use was associated with significantly greater healthcare utilization, especially non-HIV-specific healthcare visits. Relative to non-cocaine-using participants, cocaine-using individuals reported twice more non-HIV-specific healthcare visits at baseline and 2.6-fold more visits at the 6-month follow-up. Reasons for this finding are unclear. There are numerous well-characterized side effects of cocaine use, including disrupted sleep, decreased appetite, and anxiety/agitation, which may motivate healthcare engagement. Cocaine has well-documented effects on sleep architecture, including reduced total sleep time and suppressed REM sleep (Angarita et al. 2016, Schierenbeck et al. 2008), and sleep duration is shorter among individuals who co-use cocaine and cannabis compared to cocaine alone (Wheeler et al. 2021). At baseline, we found that cocaine users reported worse sleep, but that difference was not observed at 3- or 6-month follow-up, suggesting poor sleep alone is unlikely to explain more frequent healthcare visits which were present at baseline and 6-month follow-up. We found no evidence that cocaine co-use worsened appetite, anxiety, or other symptoms at any timepoint. Thus, from the lived experience perspective, more-frequent healthcare utilization may reflect broader psychosocial burdens, and unmet treatment needs (Metsch et al. 2023), rather than specific symptom burden, at least as measured in this study. National data have similarly shown that psychostimulant (particularly meth/amphetamine) use is associated with high-risk sexual behaviors, poor adherence to ART, and reduced viral suppression among PLWH (Maloney et al. 2020).

Somatic and psychiatric symptoms were generally stable across the 6-month follow-up period in the overall sample. Only poor appetite exhibited a non-significant longitudinal trend: ~17% decrease from baseline to 6-month follow-up (*p*_FDR_=0.08). The stability of somatic and psychiatric symptoms across time in this complex clinical population was unexpected, especially because significant changes in cannabis-use frequency and healthcare utilization were observed; however, symptom stability could be secondary to the fact that most of these patients had been receiving HIV care for many years.

This study has several methodological strengths, including a well-defined sample of daily cannabis users recruited from one HIV clinic and repeated clinically informative assessments over a 6-month longitudinal follow-up period. However, limitations should be noted. Viral load and CD4 cell count data were only available in a subset of subjects (*n* = 81; 68.1% of the sample). Among those participants with data, most exhibited viral load suppression (74.1%; ≤200 copies/ml) prior to baseline; however, this percentage is much lower than the UNAIDS 95% target for viral load suppression (Frescura et al. 2022), which highlights unmet need in this sample. The relatively small sample size of the Coc+ group limited our ability to detect effects of psychostimulant co-use on HIV and psychiatric symptom trajectories. Also, our reliance on self-reported measures introduces potential recall bias. Subject enrollment by recruitment method and exclusion criteria metrics were not tracked. Moreover, the study did not include a control group of individuals who did not use cannabis, with or without cocaine use, which limits our ability to isolate unique effects of cannabis vs. polysubstance use relative to abstinence. Sample demographics reflect that of PLWH in the Detroit metropolitan area (i.e., mostly male and Black/African-American) but may not generalize to other patient populations. Future studies should incorporate qualitative assessments and open-ended interviews to better understand the patient’s lived experience, as well as biomarkers, such as hair analyses for cumulative drug exposure (Becker et al. 2022) or actigraphy-based sleep assessments (Romanelli and Moore 2021) to broaden understanding of cannabis’s effects on health outcomes in PLWH. Future research should examine whether interventions such as tailored THC: cannabidiol formulations (Mboumba Bouassa et al. 2024) or behavioral strategies including contingency management to reinforce reductions in psychostimulant use and increased antiretroviral adherence (Steiner et al. 2025), could improve symptom control while minimizing healthcare burden. Finally, given the evolving legal landscape of cannabis, longitudinal studies are needed to determine how policy changes impact patient behavior and attitudes, access to standardized cannabis products, and overall health outcomes in PLWH.

In conclusion, this study offers new insights into complex relationships between longitudinal patterns of cannabis use, symptom severity, and healthcare utilization in PLWH. Whereas cannabis-use frequency was not significantly associated with HIV-related symptom relief, it was linked to HIV clinic visits, suggesting that more-frequent cannabis use (or related sequalae) may contribute to continued HIV healthcare engagement, unrelated to viral suppression. Cocaine co-use was associated with worse sleep quality, more-frequent healthcare utilization, and less-frequent cannabis use. Finally, cross-sectional associations between somatic symptoms, psychiatric symptoms, and healthcare utilization suggest an unmet need for targeted interventions among PLWH to reduce symptom burden. Future research using larger samples is needed to further investigate the interplay between cannabis use, psychostimulant co-use, and healthcare engagement, while incorporating biomarkers and other health outcomes (Metsch et al. 2023, Mboumba Bouassa et al. 2024).

## Supplementary Information

Supplementary Material 1.

## Data Availability

Data will be made available upon reasonable request.

## References

[CR1] Andreae MH, Carter GM, Shaparin N, et al. Inhaled cannabis for chronic neuropathic pain: a meta-analysis of individual patient data. J Pain. 2015;16:1221–32. 10.1016/j.jpain.2015.07.009. 26362106 10.1016/j.jpain.2015.07.009 PMC4666747

[CR2] Angarita GA, Emadi N, Hodges S, Morgan PT. Sleep abnormalities associated with alcohol, cannabis, cocaine, and opiate use: a comprehensive review. Addict Sci Clin Pract. 2016;11. 10.1186/s13722-016-0056-7. 10.1186/s13722-016-0056-7 PMC4845302 27117064

[CR3] Arfken CL. Wayne County (Detroit Area) Sentinel Community Site: Drug use patterns and trends, 2018. National Drug Early Warning System; 2018.

[CR4] Barroso J, Hammill BG, Leserman J, Salahuddin N, Harmon JL, Pence BW. Fatigue in HIV-infected people: a three-year observational study. J Pain Symptom Manage. 2010;40:69–79. 10.1016/j.jpainsymman.2009.11.325. 10.1016/j.jpainsymman.2015.02.006 PMC4492863 25701691

[CR5] Beck AT, Steer RA, Brown GK. Manual for the Beck Depression Inventory–II. San Antonio, TX: Psychological Corporation; 1996.

[CR6] Becker WC, Merlin JS, Manhapra A, et al. Cannabis use and healthcare utilization among people with chronic pain. J Gen Intern Med. 2022;37:1093–101. 10.1007/s11606-021-07119-5.

[CR7] Benjamini Y, Hochberg Y. Controlling the false discovery rate: a practical and powerful approach to multiple testing. J R Stat Soc B. 1995;57:289–300.

[CR8] Carrico AW, Shoptaw S, Cox C, et al. Stimulant use and progression to AIDS or mortality after the initiation of highly active antiretroviral therapy. JAIDS. 2014;67:508–13. 10.1097/QAI.0000000000000364. 25271387 10.1097/QAI.0000000000000364 PMC4232455

[CR9] Chesson HW, Song R, Bingham A, Farnham PG. The estimated number and lifetime medical cost of HIV infections attributable to sexually transmitted infections acquired in the United States in 2018. Sex Transm Dis. 2021;48:292–8. 10.1097/OLQ.0000000000001358. 33492098 10.1097/OLQ.0000000000001358 PMC10676007

[CR10] Compton MA. Cold-pressor pain tolerance in opiate and cocaine abusers. J Pain Symptom Manage. 1994;9:462–73. 10.1016/0885-3924(94)90203-8. 7822886 10.1016/0885-3924(94)90203-8

[CR11] Costiniuk CT, Saneei Z, Salahuddin S, et al. Cannabis consumption in people living with HIV: reasons for use, secondary effects, and opportunities for health education. Cannabis Cannabinoid Res. 2019;4:204–13. 10.1089/can.2018.0068. 31579835 10.1089/can.2018.0068 PMC6757238

[CR12] Crocker CE, Carter AJ, Emsley JG, et al. When cannabis use goes wrong: mental health side effects of cannabis use that present to emergency services. Front Psychiatry. 2021;12. 10.3389/fpsyt.2021.640222. 10.3389/fpsyt.2021.640222 PMC7917124 33658953

[CR13] Cservenka A, Racine SE, Allen MJ. Sex differences in the effects of cannabis on cognitive and affective processes. Curr Addict Rep. 2018;5:200–10. 10.1007/s40429-018-0204-8.

[CR14] Daldegan-Bueno D, Maia LO, Glass M, et al. Co-exposure of cocaine and cannabinoids and its association with biological, behavioural and health outcomes. Eur Neuropsychopharmacol. 2021;51:106–31. 10.1016/j.euroneuro.2021.06.002. 34273801 10.1016/j.euroneuro.2021.06.002

[CR15] Ferrando S, Evans S, Goggin K, Sewell M, Fishman B, Rabkin J. Fatigue in HIV illness: relationship to depression, physical limitations, and disability. Psychosom Med. 1998;60:759–64. 9847037 10.1097/00006842-199811000-00019

[CR16] Frescura L, Godfrey-Faussett P, Feizzadeh A, et al. Global HIV progress and challenges: UNAIDS estimates on viral suppression. Lancet HIV. 2022;9:e829–41. 10.1016/S2352-3018(22)00223-2.

[CR17] Fuster D, Cheng DM, Allensworth-Davies D, et al. No detectable association between frequency of marijuana use and health or healthcare utilization among primary care patients who screen positive for drug use. J Gen Intern Med. 2014;29:133–9. 10.1007/s11606-013-2605-z. 24048656 10.1007/s11606-013-2605-z PMC3889953

[CR18] Gebreyesus SH, Belay A, Berhe K et al. Prevalence and associated factors of fatigue among people living with HIV/AIDS. HIV AIDS (Auckl). 2020;12:573–82. 10.2147/HIV.S267263.

[CR19] Gonzalez-Pinto A, Alberich S, Barbeito S, et al. Cannabis and first-episode psychosis: different long-term outcomes depending on continued or discontinued use. Schizophr Bull. 2009;37:631–9. 10.1093/schbul/sbp126. 19915168 10.1093/schbul/sbp126 PMC3080669

[CR20] Gorelick DA. Cannabis use disorder. N Engl J Med. 2023;389:2267–75. 10.1056/NEJMra2212152. 38091532 10.1056/NEJMra2212152

[CR21] Greenwald MK. Mechanisms, efficacy and safety of cannabinoids for treating chronic pain. In: Balon R, Morreale M, editors. The Other Side of Cannabis: Impact on Mental and Physical Health. Washington, DC: American Psychiatric Association Publishing; 2025. pp. 305–28.

[CR22] Greenwald MK, Akcasu N, Baal P, Outlaw AY, Cohn JA, Lundahl LH. Cannabis and complementary/alternative self-treatment approaches for symptom management among African American persons living with HIV. AIDS Care. 2023;35:78–82. 10.1080/09540121.2021.1998311. 34743619 10.1080/09540121.2021.1998311 PMC9076753

[CR23] Haller VL. Long-term effects of cannabis on pain and affective symptoms. Curr Opin Behav Sci. 2024;52:101343. 10.1016/j.cobeha.2023.101343.

[CR24] Han S, Zhang Y, Yang X, et al. Exploring core mental health symptoms among persons living with HIV: a network analysis. Front Psychiatry. 2023;14. 10.3389/fpsyt.2023.1081867. 10.3389/fpsyt.2023.1081867 PMC9895861 36741117

[CR25] HIV.gov. HIV statistics overview. n.d. https://www.hiv.gov. Accessed May 24, 2025.

[CR26] Irwin MR, Archer G, Olmstead R. Fatigue and depression: biological mechanisms and clinical implications. Brain Behav Immun. 2018;73:1–10. 10.1016/j.bbi.2018.04.009.29908964

[CR27] Kipp AM, Rebeiro PF, Shepherd BE, Brinkley-Rubinstein L, Turner M, Bebawy S, Sterling TR, Hulgan T. Daily marijuana use is associated with missed clinic appointments among HIV-infected persons engaged in HIV care. AIDS Behav. 2017;21:1996–2004. 10.1007/s10461-017-1716-7. 28213820 10.1007/s10461-017-1716-7 PMC5493511

[CR28] Lee F, Jain JP, Duthely LM, Ikeda J, Santos GM. Stimulant use associated with psychosocial factors, HIV risk, and concurrent hazardous alcohol use among US adults. JMIR Form Res. 2023;7. 10.2196/45717. 10.2196/45717 PMC10472175 37590045

[CR29] Lee JY, Lee JE, Moskowitz JT, et al. An autoregressive cross-lagged model unraveling co-occurring stimulant use and HIV. Drug Alcohol Depend. 2021;225:108752. 10.1016/j.drugalcdep.2021.108752. 34144507 10.1016/j.drugalcdep.2021.108752 PMC8369386

[CR30] Mahboub N, Rizk R, Karavetian M, de Vries N. Nutritional status and dietary intake of people who use drugs: a systematic review. Nutrients. 2020;12:1–19. 10.3390/nu12020409.

[CR31] Maloney KM, Bratcher A, Wilkerson JM, Sullivan PS. Psychostimulant use, sexual risk behavior, and HIV care outcomes among men living with HIV. AIDS Behav. 2020;24:207–17. 10.1007/s10461-019-02537-4.

[CR32] Mboumba Bouassa RS, Ananga R, Ghislain M, et al. Cannabidiol–THC formulations and symptom management in people living with HIV. AIDS. 2024;38:1123–32. 10.1097/QAD.0000000000003802.

[CR33] Metsch LR, Feaster DJ, Gooden L, et al. Substance use, unmet healthcare needs, and HIV outcomes among people living with HIV. AIDS Behav. 2023;27:1234–45. 10.1007/s10461-022-03944-7.36219270

[CR34] Michigan Legislature. Michigan Medical Marihuana Act: Initiated Law 1 of 2008. § 333.26421. https://www.legislature.mi.gov/Laws/MCL?objectName=mcl-Initiated-Law-1-of-2008

[CR35] Montgomery L, Bagot K, Brown JL, Haeny AM. The association between marijuana use and HIV continuum of care outcomes: a systematic review. Curr HIV/AIDS Rep. 2019;16:17–28. 10.1007/s11904-019-00422-z. 30671919 10.1007/s11904-019-00422-z PMC6432787

[CR36] Moses TEH, Greenwald MK. History of regular nonmedical sedative and/or alcohol use differentiates substance-use patterns and consequences among chronic heroin users. Addict Behav. 2019;97:14–9. 10.1016/j.addbeh.2019.05.017. 31112911 10.1016/j.addbeh.2019.05.017 PMC6581601

[CR37] Perron BE, Bohnert K, Perone AK, Bonn-Miller MO, Ilgen M. Use of prescription pain medications among medical cannabis patients. J Stud Alcohol Drugs. 2015;76:406–13. 10.15288/jsad.2015.76.406. 25978826 10.15288/jsad.2015.76.406 PMC4440298

[CR38] Roehler DR, Hoots BE, Holland KM, et al. Trends and characteristics of cannabis-associated emergency department visits in the United States, 2006–2018. Drug Alcohol Depend. 2022;232:109288. 10.1016/j.drugalcdep.2022.109288. 35033959 10.1016/j.drugalcdep.2022.109288 PMC9885359

[CR39] Romanelli RJ, Moore DJ. Actigraphy-based sleep assessment in people living with HIV. Sleep Health. 2021;7:343–50. 10.1016/j.sleh.2021.03.005.

[CR40] Schierenbeck T, Riemann D, Berger M, Hornyak M. Effect of illicit recreational drugs upon sleep: cocaine, ecstasy and marijuana. Sleep Med Rev. 2008;12:381–9. 10.1016/j.smrv.2007.12.004. 18313952 10.1016/j.smrv.2007.12.004

[CR41] Scott W, Arkuter C, Kioskli K, et al. Psychosocial factors associated with persistent pain in people with HIV: a systematic review with meta-analysis. Pain. 2018;159:2461–76. 10.1097/j.pain.0000000000001369. 30130299 10.1097/j.pain.0000000000001369 PMC6250281

[CR42] Shelton SK, Mills E, Saben JL, et al. Why do patients come to the emergency department after using cannabis? Clin Toxicol. 2019;58:453–9. 10.1080/15563650.2019.1657582. 10.1080/15563650.2019.1657582 PMC7073292 31526057

[CR43] Spielberger CD. State-Trait Anxiety Inventory. Palo Alto, CA: Consulting Psychologists; 1989.

[CR44] Steiner JL, Shoptaw S, Reback CJ, et al. Contingency management to improve stimulant use outcomes and antiretroviral adherence among people living with HIV. AIDS Behav. 2025;29:245–56. 10.1007/s10461-024-04192-3.

[CR45] Stith SS, Vigil JM, Brockelman F, Keeling K, Hall B. Patient-reported symptom relief following medical cannabis consumption. Front Pharmacol. 2018;9:916. 10.3389/fphar.2018.00916. 30210337 10.3389/fphar.2018.00916 PMC6121171

[CR46] Swendsen J, Conway KP, Degenhardt L, et al. Mental disorders as risk factors for substance use, abuse and dependence. Addiction. 2010;105:1117–28. 10.1111/j.1360-0443.2010.02902.x. 20331554 10.1111/j.1360-0443.2010.02902.x PMC2910819

[CR47] Trickey A, Sabin CA, Burkholder G, et al. Life expectancy after 2015 of adults with HIV on long-term antiretroviral therapy in Europe and North America. Lancet HIV. 2023;10. 10.1016/S2352-3018(23)00028-0. 10.1016/S2352-3018(23)00028-0 PMC10288029 36958365

[CR48] Wagner GJ, Goggin K, Remien RH, et al. A closer look at depression and its relationship to HIV antiretroviral adherence. Ann Behav Med. 2011;42:352–60. 10.1007/s12160-011-9295-8. 21818528 10.1007/s12160-011-9295-8 PMC3226751

[CR49] Wheeler AL, Davis JM, Jacobson M, et al. Sleep duration and quality among individuals who co-use cocaine and cannabis. Drug Alcohol Depend. 2021;221:108635. 10.1016/j.drugalcdep.2021.108635. 33631551 10.1016/j.drugalcdep.2021.108635 PMC8026665

[CR50] Wrona A, Justice AC, Tate JP, et al. Cannabis use and self-reported bothersome symptoms in people with HIV. Cannabis. 2025. 10.26828/cannabis/2025/000269. 39968492 10.26828/cannabis/2025/000269 PMC11831903

[CR51] Zachary RA. Shipley Institute of Living Scale. Los Angeles, CA: Western Psychological Services; 1991.

