# Supplementary material for: Longitudinal analysis of symptoms and healthcare utilization among daily cannabis-using persons living with HIV: impact of co-occurring cocaine use
Source: J Cannabis Res. 2026 May 1;8:75. doi: 10.1186/s42238-026-00443-7 (PMC13288851; doi:10.1186/s42238-026-00443-7)
Supplement: Supplementary file 1 — Supplementary Material 1. [file 42238_2026_443_MOESM1_ESM.docx]

**Supplementary Materials To:**

**Longitudinal Analysis of Symptoms and Healthcare Utilization Among Daily Cannabis-Using Persons Living with HIV: Impact of Co-Occurring Cocaine Use**

Valden A. Denha^1,2^, Leslie H. Lundahl ^3^, Jonathan A. Cohn ^4^, Mark K. Greenwald ^3,5 ¶^, &

Eric A. Woodcock ^3,6, ¶^

^1^ Department of Population and Health Sciences, School of Public Health, University of Michigan, Ann Arbor, MI USA

^2^ Irvin D. Reid Honors College, College of Liberal Arts and Sciences, Wayne State University, Detroit, MI USA

^3^ Department of Psychiatry and Behavioral Neurosciences, Wayne State University School of Medicine, Detroit, MI USA

^4^ Department of Internal Medicine, Wayne State University School of Medicine, Detroit, MI USA

^5^ Department of Pharmacy Practice, Eugene Applebaum College of Pharmacy and Health Sciences, Wayne State University, Detroit, MI USA

^6^ Department of Pharmacology, Wayne State University School of Medicine, Detroit, MI USA

^¶^ Co-Senior Authors

**Supplemental Table 1:** FDR-corrected Pearson Correlations Among the Full Sample

|  | **Baseline Assessment** | | | | | | | | |  |
| --- | --- | --- | --- | --- | --- | --- | --- | --- | --- | --- |
|  |  | **2** | **3** | **4** | **5** | **6** | **7** | **8** | **9** |  |
| **1** | Past 90-day Cannabis Use Frequency (#) | - | - | - | - | - | - | - | - |  |
|  | *Past 90-day Healthcare Utilization* |  |  |  |  |  |  |  |  |  |
| **2** | Healthcare Clinic Visits (#) |  | - | - | - | - | - | - | - |  |
| **3** | HIV Clinic Visits |  |  |  |  |  |  |  |  |  |
|  | *Past 90-day Symptom Severity (0-3)* |  |  |  |  |  |  |  |  |  |
| **4** | Poor Appetite | - | - | - | **0.34*** | **0.28*** | - | **0.37*** | **0.31*** |  |
| **5** | Fatigue/Low Energy | - | - | - | - | **0.37*** | **0.27*** | **0.47*** | **0.39*** |  |
| **6** | Pain | - | - | - | - | - | - | **0.25*** | - |  |
| **7** | Poor Sleep | - | - | - | - | - | - | **0.41*** | **0.31*** |  |
|  | *Psychiatric Symptom Severity* |  |  |  |  |  |  |  |  |  |
| **8** | Depression (BDI-II) | - | - | - | - | - | - | - | **0.76*** |  |
| **9** | Anxiety (STAI; *state*) | - | - | - | - | - | - | - | - |  |
|  |  |  |  |  |  |  |  |  |  |  |
|  | **3-Month Assessment** | | | | | | | | |  |
|  |  | **2** | **3** | **4** | **5** | **6** | **7** | **8** | **9** |  |
| **1** | Past 90-day Cannabis Use Frequency (#) | - | - | - | - | - | - | - | - |  |
|  | *Past 90-day Healthcare Utilization* |  |  |  |  |  |  |  |  |  |
| **2** | Healthcare Clinic Visits (#) | - | - | - | **0.41*** | - | **0.29*** | **0.33*** | - |  |
| **3** | HIV Clinic Visits | - | - | - | - | - | - | - | - |  |
|  | *Past 90-day Symptom Severity (0-3)* |  |  |  |  |  |  |  |  |  |
| **4** | Poor Appetite | - | - | - | **0.29*** | - | - | **0.29*** | **0.36*** |  |
| **5** | Fatigue/Low Energy | - | - | - | - | **0.40*** | **0.51*** | **0.51*** | **0.47*** |  |
| **6** | Pain | - | - | - | - | - | **0.28*** | - | - |  |
| **7** | Poor Sleep | - | - | - | - | - | - | **0.33*** | **0.29*** |  |
|  | *Psychiatric Symptom Severity* |  |  |  |  |  |  |  |  |  |
| **8** | Depression (BDI-II) | - | - | - | - | - | - | - | **0.76*** |  |
| **9** | Anxiety (STAI; *state*) | - | - | - | - | - | - | - | - |  |
|  |  |  |  |  |  |  |  |  |  |  |
| **6-Month Assessment** | | | | | | | | | |  |
|  |  | **2** | **3** | **4** | **5** | **6** | **7** | **8** | **9** |  |
| **1** | Past 90-day Cannabis Use Frequency (#) | - | - | - | - | - | - | - | - |  |
|  | *Past 90-day Healthcare Utilization* |  |  |  |  |  |  |  |  |  |
| **2** | Healthcare Clinic Visits (#) | - | - | - | - | **0.36*** | - | **0.27*** | - |  |
| **3** | HIV Clinic Visits | - | - | - | - | - | - | - | - |  |
|  | *Past 90-day Symptom Severity (0-3)* |  |  |  |  |  |  |  |  |  |
| **4** | Poor Appetite | - | - | - | **0.48*** | - | **0.29*** | **0.34*** | **0.34*** |  |
| **5** | Fatigue/Low Energy | - | - | - | - | **0.33*** | **0.50*** | **0.57*** | **0.45*** |  |
| **6** | Pain | - | - | - | - | - | **0.30*** | - | - |  |
| **7** | Poor Sleep | - | - | - | - | - | - | **0.48*** | **0.38*** |  |
|  | *Psychiatric Symptom Severity* |  |  |  |  |  |  |  |  |  |
| **8** | Depression (BDI-II) | - | - | - | - | - | - | - | **0.71*** |  |
| **9** | Anxiety (STAI; *state*) | - | - | - | - | - | - | - | - |  |
| Note: Raw Pearson *r* values are shown whereas statistical significance evaluated after False Discovery Rate (FDR; Benjamini-Hochberg) correction: **p*<0.05, ***p*<0.01, ****p*<0.001. | | | | | | | | | | |

**Supplemental Table 2:** Longitudinal Change Score Data

| **∆ Baseline → 3 Months** | | | | | |
| --- | --- | --- | --- | --- | --- |
|  | Overall (N=96-119) | Time *p_FDR_* | Coc+ (n=25-34) | Coc- (n=71-85) | Group *p_FDR_* |
| Past 90-day Cannabis Use Freq (#) | **-40.1 (174.0)** | **0.04** | -21.0 (147.0) | -47.0 (183.2) | - |
| *Past 90-day Healthcare Utilization* |  |  |  |  |  |
| Healthcare Clinic Visits (#) | -0.4 (2.1) | - | -1.0 (2.6) | -0.3 (2.8) | - |
| HIV Clinic Visits | -0.4 (1.7) | 0.08 | -0.9 (2.4) | -0.2 (1.4) | 0.08 |
| *Past 90-day Symptom Severity (0-3)* |  |  |  |  |  |
| Poor Appetite | 0.2 (1.2) | - | 0.9 (1.8) | 0.1 (1.4) | - |
| Fatigue/Low Energy | 0.2 (1.1) | - | 0.7 (1.5) | 0.2 (1.5) | - |
| Pain | 0.1 (1.2) | - | -0.2 (1.0) | -0.1 (1.3) | - |
| Poor Sleep | 0.2 (1.2) | - | -0.6 (1.1) | -0.01 (1.2) | 0.08 |
| *Psychiatric Symptom Severity* |  |  |  |  |  |
| Depression (BDI-II) | 0.9 (8.3) | - | -3.0 (6.7) | -0.2 (8.8) | - |
| Anxiety (STAI; *state*) | 0.3 (9.5) | - | -1.6 (7.8) | 0.2 (10.0) | - |
| **∆ 3 Months → 6 Months** | | | | | |
|  | Overall (N=85-119) | Time *p_FDR_* | Coc+ (n=21-34) | Coc- (n=64-85) | Group *p_FDR_* |
| Past 90-day Cannabis Use Freq (#) | 24.2 (163.6) | - | 0.3 (123.8) | 32.0 (174.9) | - |
| *Past 90-day Healthcare Utilization* |  |  |  |  |  |
| Healthcare Clinic Visits (#) | -0.3 (2.3) | - | 1.0 (3.1) | 0.02 (1.9) | - |
| HIV Clinic Visits | **-0.4 (1.5)** | **0.02** | 0.5 (1.2) | -0.47 (2.0) | - |
| *Past 90-day Symptom Severity (0-3)* |  |  |  |  |  |
| Poor Appetite | 0.1 (1.1) | - | -0.3 (2.0) | 0.05 (1.6) | - |
| Fatigue/Low Energy | -0.02 (1.1) | - | -0.1 (1.9) | 0.2 (1.5) | - |
| Pain | -0.01 (1.0) | - | -0.3 (1.2) | 0.1 (0.9) | - |
| Poor Sleep | -0.05 (1.3) | - | 0.0 (1.3) | 0.1 (1.3) | - |
| *Psychiatric Symptom Severity* |  |  |  |  |  |
| Depression (BDI-II) | 0.9 (6.8) | - | 0.9 (5.5) | -1.5 (7.1) | - |
| Anxiety (STAI; *state*) | 0.7 (8.7) | - | -0.1 (7.1) | -1.0 (9.2) | - |

| **∆ Baseline → 6 Months** | | | | | |
| --- | --- | --- | --- | --- | --- |
|  | Overall (N=96-97) | Time *p_FDR_* | Coc+ (n=21-34) | Coc- (n=64-85) | Group *p_FDR_* |
| Past 90-day Cannabis Use Freq (#) | -8.7 (217.8) | - | -16.8 (197.4) | -5.3 (226.9) | - |
| *Past 90-day Healthcare Utilization* |  |  |  |  |  |
| Healthcare Clinic Visits (#) | 0.0 (2.3) | - | 0.1 (3.5) | -0.04 (1.6) | - |
| HIV Clinic Visits | **-0.7 (1.5)** | **0.002** | -1.2 (2.1) | -0.7 (1.7) | - |
| *Past 90-day Symptom Severity (0-3)* |  |  |  |  |  |
| Poor Appetite | 0.2 (1.0) | 0.08 | 0.04 (1.0) | -0.3 (1.0) | - |
| Fatigue/Low Energy | 0.2 (1.2) | - | 0.04 (1.3) | -0.3 (1.2) | - |
| Pain | 0.2 (1.1) | - | -0.4 (1.1) | -0.1 (1.1) | - |
| Poor Sleep | 0.1 (1.2) | - | -0.4 (1.3) | -0.03 (1.1) | - |
| *Psychiatric Symptom Severity* |  |  |  |  |  |
| Depression (BDI-II) | 1.1 (8.3) | - | -0.1 (7.5) | -1.5 (8.6) | - |
| Anxiety (STAI; *state*) | 0.9 (9.0) | - | -1.2 (7.2) | -0.7 (9.7) | - |

| Note: Mean (±1 SD) depicted. False Discovery Rate (FDR; Benjamini-Hochberg) correction applied. |
| --- |
